# Segregation of Acetylcholine and GABA in the Rat Superior Cervical Ganglia: Functional Correlation

**DOI:** 10.3389/fncel.2016.00091

**Published:** 2016-04-07

**Authors:** Diana Elinos, Raúl Rodríguez, Luis Andres Martínez, María Elena Zetina, Fredy Cifuentes, Miguel Angel Morales

**Affiliations:** Departamento de Biología Celular and Fisiología, Instituto de Investigaciones Biomédicas, Universidad Nacional Autónoma de MéxicoCiudad de México, México

**Keywords:** co-transmission, VAChT, GAD67, SPN, GABA_A_R

## Abstract

Sympathetic neurons have the capability to segregate their neurotransmitters (NTs) and co-transmitters to separate varicosities of single axons; furthermore, in culture, these neurons can even segregate classical transmitters. *In vivo* sympathetic neurons employ acetylcholine (ACh) and other classical NTs such as gamma aminobutyric acid (GABA). Herein, we explore whether these neurons *in vivo* segregate these classical NTs in the superior cervical ganglia of the rat. We determined the topographical distribution of GABAergic varicosities, somatic GABA_A_ receptor, as well as the regional distribution of the segregation of ACh and GABA. We evaluated possible regional differences in efficacy of ganglionic synaptic transmission, in the sensitivity of GABA_A_ receptor to GABA and to the competitive antagonist picrotoxin (PTX). We found that sympathetic preganglionic neurons *in vivo* do segregate ACh and GABA. GABAergic varicosities and GABA_A_ receptor expression showed a rostro-caudal gradient along ganglia; in contrast, segregation exhibited a caudo-rostral gradient. These uneven regional distributions in expression of GABA, GABA_A_ receptors, and level of segregation correlate with stronger synaptic transmission found in the caudal region. Accordingly, GABA_A_ receptors of rostral region showed larger sensitivity to GABA and PTX. These results suggest the presence of different types of GABA_A_ receptors in each region that result in a different regional levels of endogenous GABA inhibition. Finally, we discuss a possible correlation of these different levels of GABA modulation and the function of the target organs innervated by rostral and caudal ganglionic neurons.

## Introduction

Neurons use neurotransmitters (NTs) to convey their signals through chemical synapses. Most neurons operate with more than one NT, a characteristic known as cotransmission (Burnstock, 1976; Hökfelt et al., 1977). Originally, cotransmission was defined as the presence and release of one classical transmitter along with one or more cotransmitters. Later, it was reported that cotransmission could also involve the co-existence of two classical transmitters (see the book by Gutiérrez, 2009). Sympathetic ganglia were one of the first neuronal structures where the presence of the classical NTs, acetylcholine (ACh; Feldberg and Gaddum, 1934) and noradrenaline (NA; von Euler, 1946) was shown. Later, the presence of other NTs was described, like ATP (Burnstock, 1976), various neuropeptides that neuromodulate cholinergic transmission (Hökfelt et al., 1977; Dun and Karczmar, 1979; Jan et al., 1979), as well as classical transmitters like glutamate and gamma aminobutyric acid (GABA; Wolff et al., 1986; Dobó et al., 1989; Senba et al., 1991; Ito et al., 2005, 2007). In contrast to the known modulatory function of cotransmitters, the presence of two classical NTs in sympathetic ganglia does not necessarily imply a dual phenotype (excitatory or inhibitory) ganglionic synapse, since it has been reported that GABA, besides its synaptic function, has metabolic and morphogenetic actions in the superior cervical ganglion (SCG) of the rat (Wolff et al., 1987).

Early on, it was assumed that to achieve cotransmission, neurons store and release the same set of NTs from all their processes (Chan-Palay and Palay, 1984; Burnstock, 1990; Merighi, 2002). However, evidence has shown that cotransmission can be achieved not only by the co-storage and co-release of the same combination of NTs from all neuronal synaptic endings, but also by the concurrent release of NTs previously segregated to separate synaptic boutons (Sossin et al., 1990; El Mestikawy et al., 2011; Sámano et al., 2012; Zhang et al., 2015). We and others have provided evidence that sympathetic neurons segregate ACh and diverse neuropeptides to separate varicosities (Morales et al., 1995; Chanthaphavong et al., 2003; Sámano et al., 2006, 2009, 2012). We have also shown that ciliary neurotrophic factor (CNTF) potentiates the segregation of ACh and NA to separate varicosities of sympathetic neurons co-cultured with cardiac myocytes (Vega et al., 2010). Based on these findings, we have proposed that cotransmission in sympathetic synapses is achieved not only by the co-release of ACh and cotransmitters from the same bouton, but also by the concurrent release of NTs independently stored in separate presynaptic boutons (Sámano et al., 2009, 2012). Segregation represents a simpler and more direct mechanism to release distinct transmitters from single boutons. Additionally, segregation facilitates the ability of neurons to convey different information to distinct targets. The presence of two classical transmitters in sympathetic neurons, such as ACh and GABA, raises the question whether these neurons can segregate them. To explore the possibility of this segregation, in this work we analyzed the occurrence and co-occurrence of L-glutamic acid decarboxylase (GAD67, the enzyme responsible for the synthesis of GABA) and the cholinergic markers choline acetyl transferase (ChAT) or the vesicular ACh transporter (VAChT) in the soma and varicosities of sympathetic preganglionic neurons (SPN). Furthermore, we also investigated a possible role of segregation of ACh-GABA in determining GABAergic modulation of ganglionic function. We found that, in fact, sympathetic ACh-GABA dual phenotype neurons do segregate ACh and GABA to separate varicosities in the SCG of the rat. Furthermore, we demonstrated a possible role of segregation of ACh and GABA in establishing different levels of inhibitory regulation of sympathetic transmission.

## Materials and Methods

Experiments were carried out on male Wistar rats (200–250 g), which were treated in accordance with the ethical guidelines for the care and use of laboratory animals of the National Academy of Sciences of the United States and approved by our Institutional Committee for the Care and Use of Animals in the Laboratory. For all surgical procedures, rats were anesthetized with xylazine (10 mg/kg i.m.) and ketamine (90 mg/kg i.p.).

### Histological Procedures

To decentralize the ganglion, the sympathetic thoracic trunk (STT) was exposed and cut 3–5 mm caudal to the ganglion. After the surgical procedure, the animals were given postoperative care. To label SPN or ganglionic neurons, the Fluoro-Gold (FG) tracer (Fluorochrome, LLC, Denver CO) or 3% DAPI (Sigma, St Louis, MO, USA) were applied at the distal end of the STT or at the carotid nerves, respectively, using a pulled glass pipette. To block SPN axonal transport we followed the procedure used by Albuquerque et al. (1972), briefly, a catheter was inserted into the region of the atlanto-occipital junction, reaching the 6th–8th cervical spinal cord segments, and 10–16 μg colchicine (1 μg/μL in physiological saline solution) was slowly injected intrathecally. To process the SCG for immunostaining, rats were deeply anesthetized with sodium pentobarbital (125 mg/kg i.p.) after 7 days (decentralization), 3 days (retrograde labeling of ganglionic neurons) or 5 days (retrograde labeling of SPN and colchicine treatment).

For light microscopy (LM) examination, after deep anesthesia, rats were transcardially perfused with 100 mL of ice-cold phosphate-buffered saline (0.01 M PBS, pH 7.4) for 3 min, and then with 250 mL of ice-cold fixative solution (2% paraformaldehyde, 0.18% picric acid in 0.1 M PBS, pH 7.4), 100 mL for 3 min and the remaining 150 mL for 40 min. The SCG and a spinal cord segment (from C7 to T3) were dissected, postfixed overnight in the same fixative solution, and cryoprotected in sucrose solution (10%–30%, w/v). Horizontal and transverse sections of the spinal cord and longitudinal sections of the SCG (14 μm thickness) were cut using a cryostat at −20°C, recovered on Superfrost Plus slides (Electron Microscopy Sciences, Hatfield, PA, USA), and processed at room temperature in a humid atmosphere with a routine procedure for single or double immunohistochemical staining. Tissue sections were preincubated for 2 h with 10% bovine serum albumin for all immunostaining or with 10% donkey serum for GAD67. Sections were then incubated overnight with primary polyclonal antibodies directed against ChAT, VAChT, synaptophysin (Syn), methionine enkephalin (mENK), neuropeptide Y (NPY) and GABA_A_ receptor α4 subunit (GABA_A_R α4) or monoclonal anti-GAD67 diluted in 10% donkey serum (Table 1). Due to the low efficiency of the primary antibody against GAD67, the respective sections were preincubated with ImmunoDNA Retriever Citrate solution (1:20; Bio Science for the World, Santa Barbara, CA, USA) for 1 h at 70°C. Tissue sections were rinsed twice for 10 min each in PBS–Triton X-100 (0.1 M PBS, 0.3% Triton X-100), and then incubated for 2 h with an appropriate secondary antibody (Table 1). In both tissues, as a control prior to the immunostaining procedure, the primary antibodies were preadsorbed overnight at room temperature with a 10-fold molar excess of their corresponding control antigens 3.0 μM for GAD67 (Gene Tex, Irvine, CA, USA); 0.1 μM for ChAT (Chemicon, Temecula, CA, USA) and 1 μM for the rest (mENK, Sigma, St Louis, MO, USA; Syn, Abcam, Cambridge, MA, USA; GAD67 Millipore, Chemicon Billerica, MA, USA). As another control, some tissue sections were processed through all the incubation steps, but the primary antibodies were omitted. Finally, sections were coverslipped with a fluorescence mounting medium (Dako Fluorescence Mounting Medium, Dako, Santa Clara, CA, USA) and examined with an epifluorescence microscope (Nikon Eclipse E600), equipped with the appropriate filters for Alexa 488, Alexa 594 and Cy5. Selected sections with single or double labeling were further analyzed with a confocal LSM 5 Pascal Zeiss microscope equipped with an argon/krypton laser. Images were collected with a 40× objective (1.3 NA/Oil). Confocal images were obtained using at least two separate photomultiplier channels, either concurrently or in separate runs.

**Table 1:** **Antibodies used for immunohistochemistry**.

Antiserum	Type of antibody	Coupled to	Dilution	Source	Catalogue num.
**Primary**
VAChT (rat)	Goat polyclonal	–	1:100	Promega Corp, Madison, WI, USA	ab27941
ChAT (human)	Goat polyclonal	–	1:100	Millipore, Chemicon, MA, USA	AB144P
mENK (bovine)	Rabbit polyclonal	–	1:100	Millipore, Chemicon, MA, USA	AB5026
Synaptophysin (human)	Rabbit polyclonal	–	1:200	Dako Cytomation, Denmark	A0010
GAD 67 (synthetic)	Mouse polyclonal	–	1:200	Millipore, Chemicon, MA, USA	MAB5406
NPY	Rabbit polyclonal	–	1:200	Bachem, CA, USA	T4070
α4 GABA_A_	Rabbit polyclonal	–	1:200	Sigma	69169
**Secondary**
α goat IgG	Donkey	Alexa 488	1:500	Jackson ImmunoResearch Lab, Inc.	705-545-003
α rabbit IgG	Donkey	Alexa 488	1:500	Jackson ImmunoResearch Lab, Inc.	711-545-152
α mouse IgG	Donkey	Alexa 594	1:700	Jackson ImmunoResearch Lab, Inc.	715-515-150
α mouse biotinitaled			1:200	VectorLaboratories	BA-2000
Avidine conjugated HRP (ABC).			1:100	Vector Laboratories	PK-6100

For transmission electron microscopy (TEM) examination, rats were deeply anesthetized and transcardially perfused with cold sodium phosphate buffer (PB; 0.1M, pH 7.4) followed by a fixative solution (4% paraformaldehyde and 1% glutaraldehyde in PBS). After perfusion, SCG were removed, post-fixed for 2 h at 4°C in the same fixative, and transferred to PBS. Then, ganglia were permeabilized by brief exposure to liquid nitrogen followed by a 30% sucrose solution and returned to PBS. Free floating vibratome sections (30 μm thick) were immunoprocessed in cell culture (24-well) plates, rinsed in 0.1 M PBS, and incubated in 1% sodium borohydride solution for 15 min. Sections were then thoroughly washed in 0.1 M PBS, treated with 10% normal goat serum for 30 min, and incubated for 24 h with the primary antibody against GAD67, 1:100 in 0.1 M PBS containing 3% normal goat serum. The serial sections were washed three times in PBS, incubated in biotinylated horse anti-mouse IgG (1:200, Vector Laboratories, Burglingame, CA, USA) for 2 h, washed in PBS, and then treated with avidin conjugated to horseradish peroxidase (to form avidin-biotin complexes; ABC, 1:100; Vector) for 1 h. Sections were rinsed in PBS, and the peroxidase activity was revealed with 0.05% 3,3′diaminobenzidine and 0.01% hydrogen peroxide in PBS for 5 min at room temperature. Sections were block counterstained with 10% uranyl acetate for 1 h, postfixed for 1 h in 1% OsO_4_, dehydrated in graded ethanol and embedded in Epon between plastic coverslips. After overnight curing, sections were analyzed by LM and regions with clear immunolabeling were selected to be glued onto Epon blocks and sectioned using an ultramicrotome at a thickness of 3 μm for LM and 60 nm for TEM. Sections were counterstained with uranyl acetate and lead citrate, and finally examined using a Jeol 1100 TEM.

### Electrophysiological and Pharmacological Studies

Rats were anesthetized with xylazine (10 mg/kg i.m.) and ketamine (90 mg/kg i.p.), then the ganglia were rapidly excised and carefully desheathed. The preganglionic and post-ganglionic nerve roots were trimmed to a length of 3–5 mm, and the ganglia were transferred to a recording chamber (Warner Instruments, Hamden, CT, USA) and bathed with oxygenated (95% O_2_, 5% CO_2_) Krebs-Ringer Solution, pH 7.4, containing (in mm): 136 NaCl, 4 KCl, 2 CaCl_2_, 1 MgCl_2_, 1 KH_2_PO_4_, 12 NaHCO_3_, 11 glucose and 2 μM atropine. All experiments were carried out at a controlled temperature of 24.0 ± 0.5°C. For recording and stimulation, the cervical sympathetic trunk (preganglionic) and one of the two postganglionic nerves, i.e., the internal and external carotid nerves (ICN, ECN), were pulled into glass suction electrodes to maintain a seal during recording. Stimuli were applied by a Pulsar 6i Stimulator (FHC Inc., Bowdoin, ME, USA) and consisted of supramaximal square voltage pulses (9–12 V) 0.1 ms in duration at 0.2 Hz. Compound action potentials (CAPs) were recorded from the ICN and ECN, voltage traces were amplified (100×) and bandpass filtered by a differential amplifier (DP-301, Warner Instruments, Hamden, CT, USA), and digitized with a multifunction data-acquisition (PCI-DAQ) board with 16-bit A–D converter using a custom-made acquisition program written in LabView v8.6 (National Instruments, Austin, TX, USA). The basal CAP amplitude was stable for 3–4 h.

Input-output (I/O) curves were constructed by recording the amplitude of CAPs (output) obtained either in the ECN or in the ICN in response to progressively increasing stimulus intensities (input). We evaluated these curves by fitting a logistic function to the data, *V_O_* = *V_M_*/[1 + ((*V_M_*/*V_0_*)−1)×*e^−αVi^*] (Banks, 1994), where *V_O_* = output voltage, *V_M_* = maximal response, *V_0_* = initial response (in our case ≈ 0.005), *V_i_* = input voltage, and coefficient α, related to the slope. We obtained the input value at which the half response occurred by interpolating from the graphs.

To evaluate GABA_A_R sensitivity we determined IC_50_ from dose-response curves, GABA (Sigma Chemical Co, St Louis, MO, USA) was prepared fresh in Krebs-Ringer solution. To test the effect of GABA, control CAPs were recorded during 3–5 min, then GABA was administered at the concentrations indicated and maximum inhibition was determined. After that, the preparation was washed and once the CAP control amplitude was reestablished, we waited 30 min approximately before a new GABA concentration was tested in the same manner, no more than two GABA concentration were tested in each ganglion at random order.

To evaluate regional differences in the desensitization rate to GABA, and in the sensitivity of GABA_A_R to the competitive antagonist picrotoxin (PTX) we compared the ECN and ICN responses as follow: first, we administered 250 μM GABA and compared the rate of desensitization from maximal inhibited responses in ICN and in ECN. To investigate GABA_A_R-sensitivity to PTX, we administered 20 μM PTX and 5 min later 250 μM GABA, we determined the level of GABA inhibition obtained in presence of PTX. Sensitivity to this GABA antagonist was also tested in ganglionic plasticity using submaximal stimulation, we applied a 20 Hz, 20 s train to induce a post-train potentiation, then we administered PTX 50 μM and repeated the train. To compare the potentiation we determined the response amplitude at 30 and 60 min, and the area under the post-train curve in both nerves, ICN and ECN.

### Sampling, Assessing Colocalization, and Statistics

Each ganglion was longitudinally sectioned throughout the entire length of its mediolateral axis (ca. 800 μm) to produce 40–50 slices. We sampled the tissue by collecting at least five slices at three depths (140–210, 350–420, and 560–630 μm from the edge of the ganglion) and sorted the slices onto different slides. For each ganglion, we selected a slice at random and explored its whole area by scanning with a confocal LSM 5 Pascal Zeiss microscope. For each image, we selected a single confocal plane. After virtual slice reconstruction, using the Metamorph image analysis system (v. 7.5.6; Universal Imaging Corporation, Molecular Devices, Downingtown, PA, USA), we removed out of focus blur by means of deconvolution functions. We then identified the specific labels by selecting puncta optical density (OD) that surpassed the negative staining background level (i.e., OD > background mean + 2 SD). We assessed the number of overlapping pixels for each marker in double-labeled varicosities. For GAD67 and Syn labels, colocalization is presented as the ratio of the percentage of fibers coexpressing the two labels relative to the percentage of fibers expressing one marker. Thus, a ratio of one corresponds to complete colocalization, whereas a ratio of zero represents completely independent localization. Segregation of the two labels was expressed as the percentage of varicosities expressing a given marker (e.g., GAD67) that did not show staining for a second marker (e.g., VAChT). Thus, 100% segregation indicates that none of the varicosities in a double-labeled section expressed both labels, while 0% denotes that all varicosities expressed both labels. We used four rats to assess immunolabeling of GABA_A_R α4 and three animals for each retrograde transport label. Five animals were used for all the others immunolabeling experimental groups. For electrophysiological experiments seven rats were used for the I/O curves and five for the dose-response curves.

To identify through which carotid nerve (ICN or ECN) ganglion neurons project their processes to their targets, we applied different tracers in each of the two carotid nerves, and determined where the differentially marked ganglionic neurons were located. In this way, we divided the ganglion into two regions, one occupied by neurons retrogradely labeled through the ICN and the other by the ECN. Then, in another series of experiments combining GAD67 immunostaining and retrograde labeling, we defined by which nerve the neurons densely contacted by GABA (DCG) preferentially project their processes.

To quantify SPN cell bodies expressing GAD67 and ChAT in the spinal cord, we sectioned it transversally at the levels of the exiting nerve roots or longitudinally, recovering all the tissue slices. We counted the number of cell bodies positive for either GAD67 or ChAT, or for the two markers (colocalization) in the intermediolateral nuclei (IMLn).

Data are expressed as mean ± SEM. The significance level for differences between the means was evaluated either with an independent Student’s *t*-test or, in the case of the distribution of co-localization along the ganglionic regions, with an independent one-way ANOVA followed by the Tukey *post hoc* test. The significance level was set at *P* < 0.05.

## Results

### Presence of GAD67 in Perikarya and Varicosities of SPN

**Figure 1:**
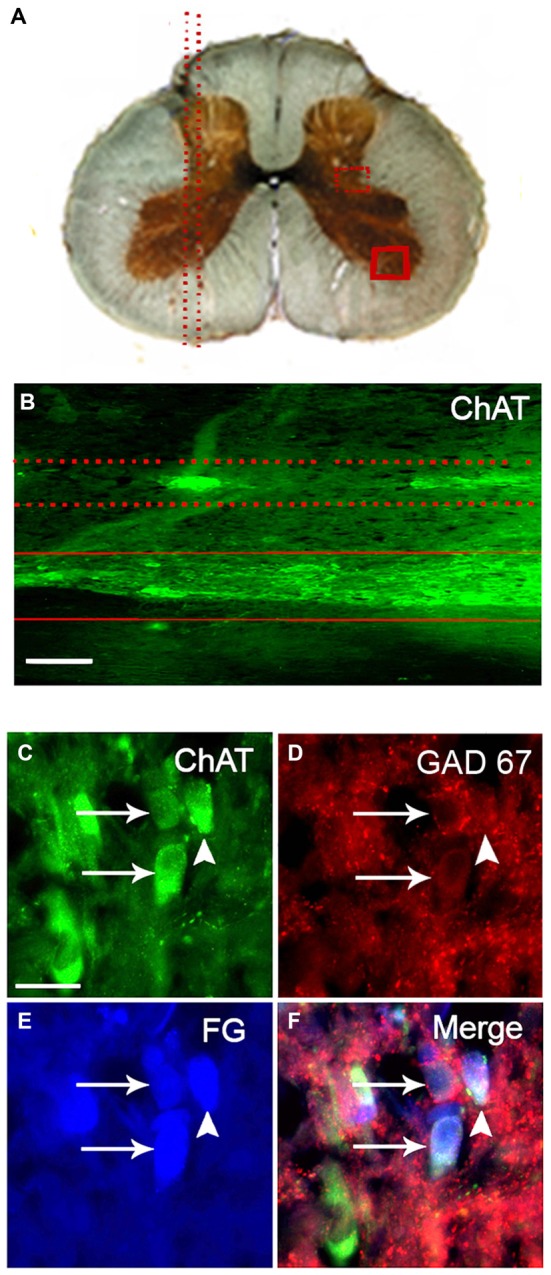
**Practically all GAD67-containing cell bodies of retrogradely labeled sympathetic preganglionic neurons (SPN) coexpress ChAT. (A)** Drawing of a transverse section of the thoracic spinal cord indicating the orientation of longitudinal sections (parallel dotted lines in left) and the location of the intermediolateral nuclei (IMLn; square of dotted lines) and a region of the ventral horn (square of solid lines) in the right. **(B)** Micrograph of a longitudinal section of spinal cord immunostained for ChAT showing the location of immunopositive SPN in the IMLn (between dotted lines) and motoneurons in the ventral horn (between solid lines). **(C–F)** Micrographs of a single transverse section of spinal cord simultaneously immunostained for ChAT **(C)**, GAD67 **(D)**, retrogradely labeled with Fluoro-Gold (FG; **E**) and the merged image **(F)**. The two GAD67-IR cell bodies depicted were positive for ChAT (arrows). There is a third ChAT-positive neuron negative for GAD67 (arrow head). FG labeled the three neurons with different levels of intensity. Scale bar 20 μm.

Considering that GAD67 is ten times less expressed in SPN than cholinergic markers, to immunolabel this enzyme in somata of the SPN we used colchicine to block the axonal transport (Albuquerque et al., 1972). Thus, in the spinal cord of colchicine treated rats, immunoreactivity to GAD67 (GAD67-IR) was detected in the cell bodies of ovoid neurons (ca. 20 μm of the major axis), within and around IMLn. This morphology and location correspond to sympathetic preganglionic neurons (SPN; Deuchars and Lall, 2015). Almost all of these putative preganglionic GAD67-IR neurons were retrogradely labeled with FG, confirming their preganglionic identity (Figure 1). Besides these immunopositive neurons, abundant GAD67-IR staining was found in varicosities surrounding SPN (Figures 1D,F).

**Figure 2:**
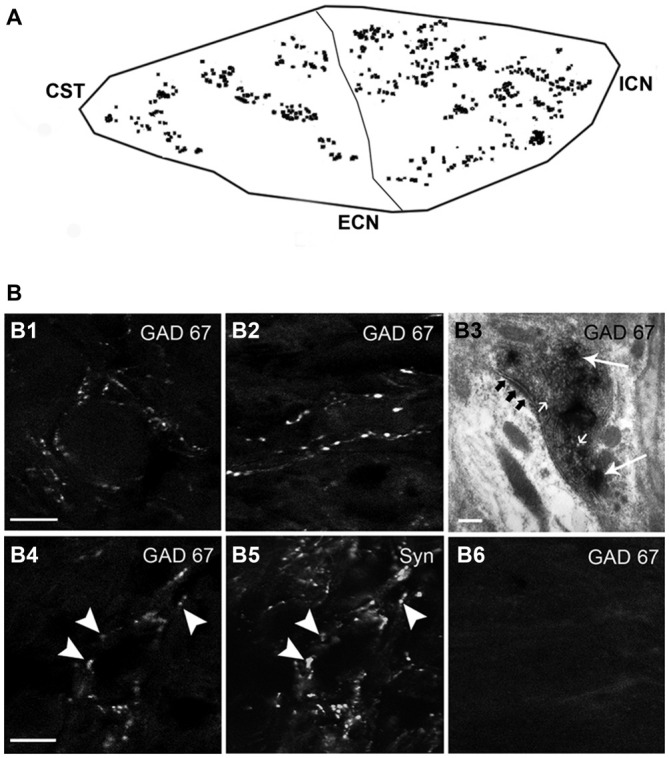
**GAD67-containing SPN varicosities exhibit a rostro-caudal gradient. (A)** Outline of the regional distribution of GAD67-containing SPN varicosities, (79 ± 6% in rostral vs. 21 ± 3% in caudal; *P* < 0.02) CST, cervical sympathetic trunk; ICN and ECN, internal and external carotid nerves. **(B)** Micrographs of superior cervical ganglion (SCG) sections immunolabeled for GAD67 **(B1–B4, B6)** and Syn **(B5)**. GAD67-containing varicose fibers exhibit two forms of local organization: concentric, encircling ganglionic principal neurons (80 ± 2%; **B1**) and interstitial in the neuropil alongside neuronal cell bodies (20 ± 3%; **B2**). Electron microscopy analysis reveal that the GAD67-IR varicosities show the characteristic features of presynaptic boutons, i.e., small clear vesicles (small arrows), large dense core vesicles (arrows), mitochondria and a presynaptic active zone (black arrows; **B3**). Practically all GAD67-containing varicosities were also immunoreactive to Syn **(B4,B5)**. Denervation of the ganglia removed all GAD67 staining, indicating its preganglionic origin **(B6)**. Scale bars 20 μm and 150 nm in **(B3)**.

As expected, some of the axon fibers of SPN arriving into the SCG were GAD67 immunopositive. Thus, GAD67-IR was found in fibers and varicosities unevenly distributed along the ganglia; we found that GAD67-IR was present in a rostro-caudal gradient (79 ± 6% in rostral vs. 21 ± 3% caudal; *P* < 0.02; Figure 2). According to its pattern we classified GAD67-IR varicosities into two types: most of them (80 ± 2%) formed concentric varicose fibers tightly encircling some ganglionic principal neurons (Figure 2B1), while the remaining 20 ± 3% of the GAD67-IR varicose fibers were long interstitial fibers within the neuropil alongside neuronal cell bodies (Figure 2B2). We also found a population of neurons densely contacted by concentric GAD67-IR varicosities. These neurons, termed as DCG neurons, could correspond to those described by Wolff et al. (1989) and by Ito et al. (2007).

To explore the origin and synaptic nature of GAD67-IR puncta, we denervated the SCG by full transection of the STT that carry on the axons of sympathetic preganglionic neurons, and performed double immunostaining for GAD67 and for Syn, a synaptic marker. We found that denervation removed all GAD67- and Syn-IR puncta, indicating their preganglionic origin (Figure 2B6). By TEM examination, we further demonstrated that GAD67-IR structures display all the ultrastructural features of presynaptic boutons, i.e., a cytoplasmic limiting membrane, small clear vesicles, large dense core synaptic vesicles, mitochondria, a presynaptic active zone and postsynaptic density. The TEM images clearly showed that GAD67-IR was present mainly on small clear vesicles, although some large dense core vesicles were also labeled (Figure 2B3). Double GAD67-Syn immunolabeling showed considerable co-localization of these two markers; 80 ± 3% of GAD67-containing varicosities also expressed Syn (Figures 2B4,B5). These data confirmed the preganglionic source and synaptic nature of the GAD67 immunopositive puncta detected in the SCG.

### Principal Ganglionic Neurons Express GABA_A_ Receptor α4 Subunit

**Figure 3:**
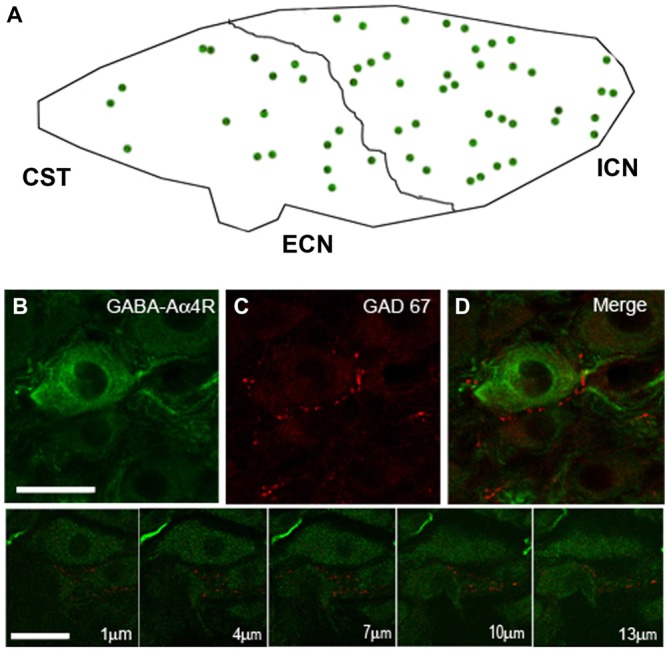
**The SCG express immunoreactivity for the GABA_A_ receptor α4-subunit (GABA_A_R α4) with a rostro-caudal gradient. (A)** Regional distribution of GABA_A_R α4; 60 ± 1% in the rostral and 40 ± 1% in caudal neurons (*P* < 0.01). **(B)** Micrographs of SCG sections showing immuno-staining for GABA_A_R α4 and GAD67 **(C)**. The pictures shown in the bottom line correspond to a series of confocal images taken through the *Z*-axis (1 μm optical section). GABA_A_R α4 was detected on the surface and in the cytoplasm of ganglionic principal neurons. A close match between GABA_A_R α4-containing neurons and GAD67-containing varicosities was frequently observed **(D)** Scale bar 20 μm.

Considering the heterogeneous distribution of ganglionic GAD67-IR varicosities in the SCG, we wondered whether GABA_A_R-IR follows a similar topographical distribution. We found that 56 ± 2% of the principal ganglionic neurons were immunoreactive to GABA_A_ receptor α_4_ subunit; we also detected immunostaining in some neuronal processes that did not correspond to preganglionic fibers since they remained after ganglionic decentralization. Regarding its topographical distribution, like GAD67-IR, GABA_A_R α4-IR also showed a rostro-caudal gradient; thus, 60 ± 1% of positive neurons were located in the rostral pole, and the remaining 40 ± 1% was located in the caudal region (*P* < 0.01). Often, we found a close correspondence between GAD67-containing varicosities and GABA_A_R α4-expressing neurons; for example, DCG neurons usually express GABA_A_R α4-IR (Figure 3).

### VAChT is Often Segregated from GAD67-Containing Varicosities

**Figure 4:**
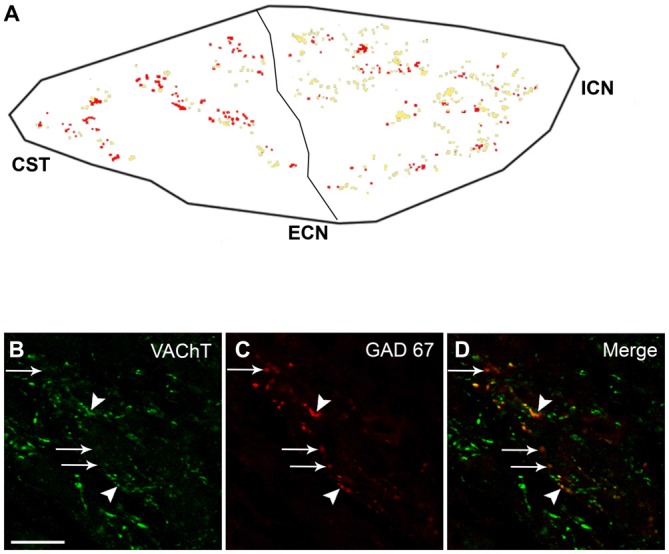
**With a caudo-rostral gradient, VAChT is segregated from preganglionic GAD67-containing varicosities. (A)** Regional distribution of segregation of VAChT from GAD67-containing varicosities; the co-occurrence of VACh and GAD67 is shown in yellow, while GAD67-containing varicosities lacking VAChT are shown in red. A greater level of segregation can be noted in the caudal region, 55 ± 5% vs. 25 ± 4% in rostral region (*P* < 0.0006). **(B–D)** Micrograph of SCG sections double immunostained for VAChT and GAD67, showing the co-occurrence of VAChT and GAD67 (yellow dots, head arrows) as well as the segregation of VAChT from GAD67-containing varicosities (red dots, arrows). Scale bar 20 μm.

To study the possible co-occurrence of GAD67 with the cholinergic markers ChAT and VAChT, we immunolabeled ChAT and GAD67 in the spinal cord and VAChT and GAD67 in the SCG. We found that, in the spinal cord, all GAD67-IR cell bodies retrogradely labeled with FG also expressed ChAT (Figure 1). In contrast with these results, in the SCG, we found that 44 ± 5% of the varicosities expressing GAD67-IR lacked VAChT-IR (Figure 4). The discrepancy between the almost complete coexpression of GABAergic and cholinergic markers in preganglionic cell bodies and the predominantly independent location in separate varicosities cannot be explained by a simple random distribution. Rather, it suggests that SPN actively segregate the classical transmitters GABA and ACh into separate varicosities. We did not find differences in the degree of VAChT segregation in the two types of GAD67-containg varicosities. However, we detected a marked regional difference in the segregation of VAChT and GAD67. Thus, 55 ± 5% of the GAD67-IR varicosities in the caudal region lacked VAChT immunoreactivity, whereas only 25 ± 4% of the GAD67-IR varicosities in the rostral region showed segregation (*P* < 0.0006). Consequently, rostral neurons mainly receive innervation from varicosities storing both transmitters, GABA and ACh, while neurons in the caudal region are preferentially innervated by GABA-containing varicosities lacking ACh.

### GAD67-IR Varicosities do not Contain Neuropeptide mENK, Another Inhibitory Ganglionic Cotransmitter

**Figure 5:**
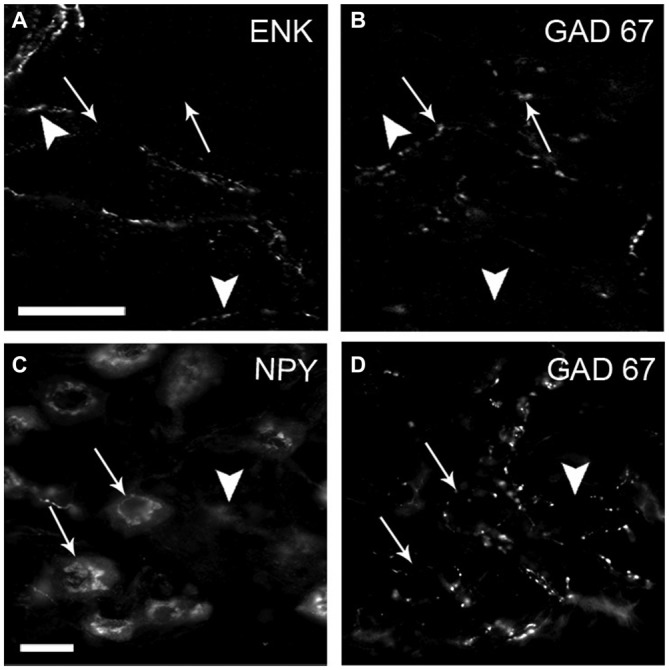
**The inhibitory neuropeptide mENK and GAD67 are stored in distinct varicosities.** Most densely contacted GABA (DCG) neurons express neuropeptide Y (NPY). Micrograph of SCG sections double-immunostained for mENK **(A)** and GAD67 **(B)** and for NPY **(C)** and GAD67 **(D)**. Immunoreactivity of mENK (head arrows) and GAD67 (arrows) was detected in separate varicosities; less than 10% of co-occurrence was detected. Two of three DCG neurons shown are positive for NPY (arrows). Scale bar 20 μm.

Taking into account the known inhibitory effect of GABA on ganglionic transmission (Adams and Brown, 1975; González-Burgos et al., 1994) we explored whether GABAergic preganglionic varicosities could store another inhibitory cotransmitter such as mENK (Zhang et al., 1995). In the double immunostaining for GAD67 and mENK, we found that most of the GAD67-containing varicosities did not contain mENK (93 ± 4%; Figures 5A,B). This absence of GAD67 and mENK co-occurrence suggests that SPN exert their inhibitory actions on ganglionic transmission through different NTs and mechanisms.

### DCG Neurons are Preferentially Located in the Rostral Region and Project Mainly through the ICN

**Figure 6:**
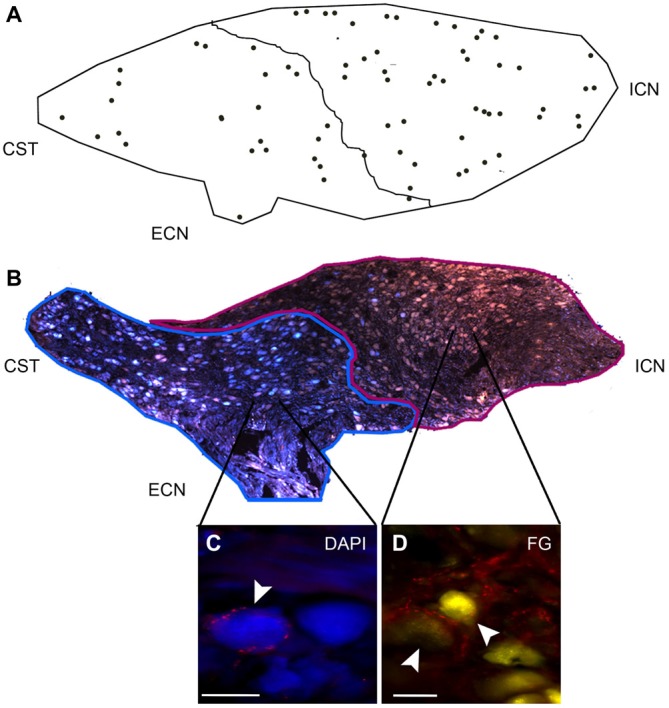
**DCG neurons show a rostro-caudal gradient. Rostral and caudal neurons project through the ICN and ECN, respectively. (A)** DCG neurons are distributed 67 ± 2% in rostral and 33 ± 4% in caudal region. **(B)** Image reconstruction of a ganglionic section showing neurons retrogradely and simultaneously stained with Fluoro-Gold (FG; yellow-brown) through the ICN and with DAPI through the ECN (blue). The former are mainly located in the rostral zone, while the latter are located in the caudal region. **(C,D)** Micrographs of SCG sections immunostained for GAD67 and retrogradely labeled with DAPI **(C)** and with FG **(D)**. Scale bar 20 μm.

In an attempt to postulate a likely role of GABA in sympathetic function, we characterized DCG neurons. Like Wolff et al. (1989), who described a rostro-caudal distribution of RIG neurons, we detected a rostro-caudal gradient of DCG neurons. We found that 58% of these neurons were located in the rostral pole, while the remaining 42% were found in the caudal region (Figure 6). To determine the ganglionic exit of these two populations, we retrogradely labeled them via the ICN and the ECN. In agreement with previous data (Bowers and Zigmond, 1979; Flett and Bell, 1991), we found that rostral ganglionic neurons project most of their axons through the ICN, while the neurons in the caudal pole project though the ECN (Figure 6B). Consequently, 67 ± 2% of rostral ganglionic DCG neurons send their axons through the ICN. We further characterized these neurons and found that, regardless their ganglionic location, most of them (63 ± 6%) were positive for NPY (Figures 5C,D). In summary, most DCG neurons are rostral, NPY-IR and send their axons through the ICN. According to these characteristics, they may correspond to ganglionic vasomotor neurons described elsewere (Gibbins, 1991; Headley et al., 2005; Li and Horn, 2006).

### A Likely Functional Role of the Regional Variation in GAD67-IR and VAChT-GAD67 Segregation

**Figure 7:**
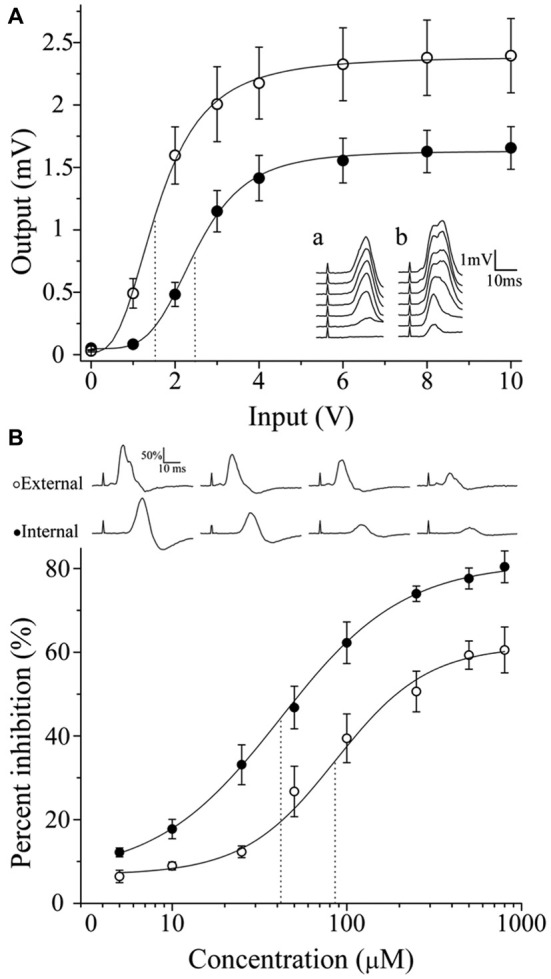
**Regionalization of synaptic transmission and GABA sensitivity in the SCG.** According to their location, neurons exhibit different activation levels and responsiveness to exogenous GABA. **(A)** Input-output curve of ganglionic transmission recorded in the ECN (empty circles) and in the ICN (filled circles). Stimuli of similar amplitude evoked a greater response in the ECN than in the ICN. Insets show a set of compound action potentials (CAPs) evoked by each input intensity tested, recorded in the ICN (a) and in the ECN (b). **(B)** Dose-response curve of the exogenous GABA effect on ganglionic transmission. Similar concentrations of GABA induced greater inhibition of CAPs recorded in the ICN. The IC_50_ values were 41.4 ± 5.5 and 86.3 ± 21.1 μM for the ICN and ECN (*P* = 0.017). The sequences of CAPs records with different levels of GABA inhibition are shown.

To determine if there is a correlation between the regional distribution of GABA-IR and classical transmitter segregation with ganglionic transmission, we recorded I/O curves of postganglionic compound action potentials (CAPs) in the ICN and the ECN to assess ganglionic transmission in the rostral and caudal areas, respectively. These curves reflect the number of responsive fibers lying in each postganglionic nerve activated at various stimulus intensities; the curve shifts when the strength of synaptic transmission is changed (Johnston and Wu, 1995). It is clearly seen in Figure 7A that neurons projecting through ECN are activated at lower voltages than those projecting through ICN, reaching higher output values. Thus, at 1 V of input, the ECN output was 0.5 ± 0.1 mV, whereas in the ICN there was a minimum response, i.e., 0.08 ± 0.01 mV (*P* = 0.004). Similarly, V_0.5_ was significantly different (1.7 ± 0.1 V for ECN vs. 2.6 ± 0.2 V for ICN; *P* = 0.001).The parameter α (related to the slope) was not significantly different (1.9 ± 0.2 for ECN and 1.8 ± 0.1 for ICN; *P* = 0.6; Figure 7A). These results indicate that ganglionic transmission through caudal neurons and leaving the ganglia through the ECN is stronger than transmission across rostral neurons.

In accordance with the strength of ganglionic transmission, the administration of exogenous GABA induced greater inhibition in the ICN than in the ECN. Thus, 800 μM GABA produced 61.5 ± 4.6 and 81.3 ± 4.5% inhibition in the ECN and ICN transmission, respectively. The IC_50_ values were 41.4 ± 5.5 and 86.3 ± 21.1 μM for the ICN and ECN, respectively (Figure 7B; *P* = 0.017).

### Functional Correlation of Regional Differences in GABA_A_R Types

Considering that different GABA_A_R types are associated with two modes of GABA inhibition described in the central nervous system (Farrant and Nusser, 2005; Lee and Maguire, 2014), it is valid to assume that regional differences in ganglionic transmission efficiency can be correlated with differential distribution of the GABA_A_R. To explore this possible correlation, we compared: the desensitization rate of GABA_A_R to exogenous 250 μM GABA; the PTX antagonistic effect on the inhibition induced by 250 μM GABA; and the effect of PTX on submaximal potentiation; in rostral vs. caudal regions.

**Figure 8:**
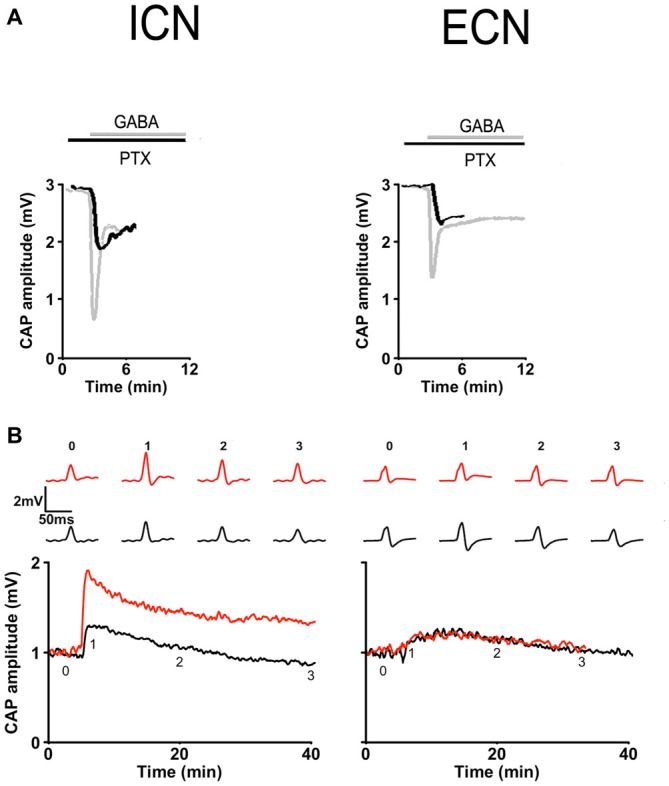
**Regional differences in GABA_A_ receptor responses in basal transmission and plasticity. (A)** Basal transmission. Time course of CAP amplitudes in response to 250 μM GABA recorded in presence of 20 μM picrotoxin (PTX) in ICN (left) and ECN (right) showing that this drug (black) antagonized in minor extent GABA inhibition in the ICN than in ECN. Gray traces depict GABA inhibition recorded in the absence of PTX. **(B)** Plasticity. Time course of potentiation evoked by a submaximal stimulation, 20 Hz, 20 s train, recorded in ICN (left) and ECN (right). In control conditions (black) and in the presence of 50 μM PTX (red). PTX disclosed a LTP in ICN but did not in ECN.

We found different desensitization rates to exogenous GABA in the ECN and ICN. (ECN: 16.1 ± 3.1 min; ICN: 2.3 ± 0.8 min; *P* < 0.001). Regarding the effect of PTX on GABA inhibition there was a significant difference in the response obtained in ECN and ICN, thus, 20 μM PTX blocked 61.2 ± 5.0% of GABA inhibition in the ECN, whereas blocked 80.8 ± 4.4% in the ICN (*P* = 0.01; Figure 8A). We tested the effect of 50 μM PTX on a submaximal potentiation and found, as González-Burgos et al. (1997), that with this protocol in control condition a small potentiation arose in rostral region that lasted less than 30 min (area under the curve = 3.9 ± 1.1 a.u.), and that 50 μM PTX increased significantly this response to become a long term potentiation lasting 60 min approximately (LTP; area under the curve = 25.7 ± 6.9 a.u.; *P* < 0.01). Similarly, in caudal region LTP was not evoked with the submaximal 20 Hz, 20 s stimulation protocol (area = 3.8 ± 1.2 a.u.), but in contrast with rostral region, 50 μM PTX did not disclose LTP (area = 3.2 ± 1.4 a.u.; Figure 8B). These regional differences in sensitivity to GABA and PTX, both in basal transmission and in synaptic plasticity, strongly suggest the presence of different GABA_A_ receptors type in caudal and rostral ganglionic neurons. It is likely that extrasynaptic receptors were prevalent in the rostral region.

## Discussion

In the present study, we demonstrate that cholinergic/GAB Aergic sympathetic preganglionic neurons *in vivo* segregate ACh and GABA into separate varicosities. We found that preganglionic GAD67-IR and ganglionic somatic GABA_A_R α4 subunit-IR showed a rostro-caudal gradient, whereas ACh-GABA segregation showed the opposite caudo-rostral gradient. These heterogeneous distributions of GABAergic innervation, GABA_A_R α4 subunit and segregation level correlate with regional differences in ganglionic transmission strength and GABA effects. Thus, synaptic transmission in the caudal region, where GABA and its receptors are expressed to a lesser extent and ACh and GABA are more segregated, is stronger than in the rostral region. Furthermore, exogenous GABA and the GABA antagonist PTX exert greater inhibition and larger effect on rostral neurons.

We have previously demonstrated the segregation of ACh and NA in separate varicosities of sympathetic ganglionic neurons co-cultured with cardiomyocytes (Vega et al., 2010). Herein, we present evidence of segregation *in vivo* of two classical sympathetic transmitters, ACh and GABA, in intact ganglia. Our data indicate that, like CNS neurons (Hattori et al., 1991; Sulzer and Rayport, 2000; Nishimaru et al., 2005; Kawano et al., 2006; Dal Bo et al., 2008; Shutoh et al., 2008; Amilhon et al., 2010; Kudo et al., 2012; Zhang et al., 2015), sympathetic neurons have the ability to sort classical NTs into separate varicosities in the SCG. Ito et al. (2007) detected preganglionic bouton-like GAD67-IR in the rat SCG, and using double-immunostaining they reported, contrary to our results, complete co-localization of VAChT with GAD67. However, in the corresponding images (Figure 4), it can be observed that some GAD67-containing varicosities lack VAChT, suggesting some degree of segregation of VAChT and GABA.

Segregation of transmitters may allow neurons to exert each transmitter function separately. For example, in the retina, it has been shown that starburst amacrine cells process complex visual signals using ACh and GABA independently. These neurotransmitters are released differentially in a Ca^2+^ level-specific manner, suggesting that the two transmitters are released from different vesicle populations (Lee et al., 2010). These authors did not exclude the possibility that ACh- and GABA-containing vesicles can be segregated and released from different presynaptic endings of single axons of amacrine cells.

The presence of GABA immunoreactive-like fiber varicosities in sympathetic ganglia has been previously reported (Kása et al., 1988; Dobó et al., 1989, 1990, 1993; Wolff et al., 1989, 1993; Párducz et al., 1992). We confirmed the presence of GAD67-IR cell bodies, fibers and varicosities of SPN as described by Ito et al. (2007). Similar to the previous work of Wolff et al. (1989), showing richly innervated GABA (RIG)-neurons, and that of Ito et al. (2007) describing neurons surrounded by GAD67-IR basket varicosities, we detected a similar set of neurons that we have called DCG. We confirmed a preferential rostral location of this type of neurons, and also found that DCG neurons are larger, express NPY and mainly project their axons through the ICN. According to these features, it is probable that they correspond to vasomotor neurons (Gibbins, 1991; Li and Horn, 2006).

In addition to confirming the presence of GAD67-IR fiber varicosities, we detected the immunoreactive expression of the GABA_A_R α4 subunit mainly within principal ganglion neurons and in some neuronal processes that did not correspond to preganglionic fibers. These results contrast with the report of Amenta et al. (1992) who detected 3H-muscimol binding sites in the rat SCG, primarily accumulated in the neuropil rather than within ganglion neurons. Likewise, the presence of presynaptic GABA_A_ and GABA_B_ receptors was inferred by pharmacological studies (Farkas et al., 1986). One possibility of these discrepancies is that other subunits different to GABA_A_R α4 are expressed in presynaptic sites; another explanation is that the level of presynaptic GABA_A_R α4 expression was below the immunostaining detection threshold. The presence of GABA_A_R β2/3 subunits in the rat major pelvic ganglion was demonstrated using immunohistochemistry by Park et al. (2006). The presence of mRNAs for at least 12 subunits of the GABA_A_R has been described in the rat SCG (Liu and Burt, 1999).

Wolff et al. (1993) suggested that some of the GABA-containing fibers in the SCG are not preganglionic, but they belong to GABAergic neurons located in the thoracic trunk or in other lower sympathetic ganglia. Accordingly, these authors proposed the existence of a GABA sympathetic interganglionic feed-forward inhibition system (Wolff et al., 1993). Our data, along with those of Ito et al. (2007), do not support this proposal, because denervation removed all the GAD67-IR in the SCG. On the other hand, like Párducz et al. (1992) and Dobó et al. (1993), we found that preganglionic SPN varicosities store GABA and the inhibitory peptide mENK separately.

We wondered whether the different degrees of ACh and GABA segregation, the regional distribution of GAD67-IR varicosities, and GABA_A_R α4 subunit expression could be related to the level of GABA inhibition of ganglionic transmission. We indeed found that the greater presence of preganglionic GABAergic varicosities and GABA_A_R α4 in the rostral region along with a low level of ACh-GABA segregation is correlated with weaker transmission and greater inhibition by exogenous GABA. In contrast, within the caudal region, where GABA and its receptors are less prevalent, along with a large degree of ACh and GABA segregation, stronger transmission and less GABA inhibition was detected. In accordance with these data, Li and Horn (2006) found that low-threshold (i.e., more excitable) neurons prevail in caudal region than in rostral region.

A relevant issue to be addressed is the possible functional role of the regional differences in ACh-GABA segregation. Both are fast action transmitters, stored in synaptic small clear vesicles; however, ACh is the main excitatory transmitter of the ganglionic synapse, whereas GABA is an inhibitory neurotransmitter of ganglionic transmission. Two types of GABA inhibition have been proposed, depending on receptor localization: if GABA_A_R are located at synaptic contacts, they will produce a transient or phasic inhibition, whereas if they are sited extrasynaptically they will produce a broad and long- lasting inhibition, termed tonic inhibition (Farrant and Nusser, 2005). In rostral region, where we found less segregation, ACh and GABA are largely co-stored in the same varicosity, and therefore will be released together regardless of the stimulation frequency. This co-store and coincident release of ACh and GABA, besides the larger presence of GABA and GABA_A_R in rostral region, could result in a stronger and more efficient GABA inhibition of cholinergic synaptic transmission. It can be also expected that rostral region would have the high-affinity extrasynaptic receptors that evoke tonic strong inhibition. On the contrary, in caudal region where we found greater segregation, and less presence of GABA and its receptors, the independent and low GABA release could induce less inhibition or even other types of GABA function. This low inhibition could be also caused by a preferential presence of low-affinity synaptic GABA_A_R in this ganglionic region. Different types of GABA inhibition have been reported in regions of the central nervous system, such as the hippocampus, cortex and cerebellum, where GABA exerts either phasic or tonic inhibition (Farrant and Nusser, 2005).

To test our hypothesis that rostral neurons preferentially contain extrasynaptic GABA_A_R, we searched specific characteristics that define synaptic and extrasynaptic GABA_A_R, such as desensitization rate and sensitivity to GABA and to antagonists like PTX. We found that rostral neurons, recorded through ICN, exhibit, a higher sensitivity to GABA and to PTX, thus this drug blocks more the inhibitory effect of GABA on basal transmission and discloses a LTP with submaximal stimulation in rostral neurons. According to the higher affinity of extrasynaptic GABA_A_ receptors to GABA and PTX (Semyanov et al., 2003; Farrant and Nusser, 2005; Lee and Maguire, 2014), our electrophysiological and pharmacological evidences provide further support to our proposal that rostral neurons contain a larger proportion of extrasynaptic receptors. Unexpectedly we found faster GABA desensitization on rostral neurons.

Finally, an important open question to be answered is the physiological role of different types of GABA inhibition on ganglionic function. It is possible that rostral neurons (including DCG neurons) positive for NPY and receiving more GABA innervation through varicosities preferentially co-storing ACh and GABA, correspond to vasomotor neurons (Li and Horn, 2006). These rostral neurons reach brain vasculature and blood vessels of rostral head, structures associated with the eye as well as pineal gland (Flett and Bell, 1991); likely, they would require a strong and tonic GABA inhibition to regulate better the vasomotor tone. On the other hand, caudal neurons, largely negative for NPY, that show less GABA innervation through varicosities containing GABA alone, they would correspond to the secretomotor type (Gibbins, 1991; Grkovic and Anderson, 1995; Li and Horn, 2006). These caudal neurons innervate the vasculature of the mouth and jaw, and the skin overlaying the caudal head, and the salivary and thyroid glands (Flett and Bell, 1991; Grkovic and Anderson, 1995); likely, they would require less GABA inhibitory modulation to exert a loose regulation of such targets. It is also feasible that GABA in the caudal region could exert another function instead of inhibition.

## Author Contributions

DE: main responsible for the experimental work, acquisition and analysis of the data; contributed to the conception and design of the work, also contributed to drafting the work and revising it. RR: responsible for experimental work, acquisition, analysis and interpretation of the electrophysiological data. LAM: responsable for electrophsyiological and pharmacological studies on GABA_A_ receptors, and the acquisition and analysis of data from those studies. MEZ: responsible for the experimental work. FC: great contribution to the conception and design of the work also contributed to the analysis and interpretation of data, drafting and revising it critically for important intellectual content. MAM: main responsible of the conception and design of the work, interpretation of data, drafting the work, writing the final manuscripts. DE, RR, LAM, MEZ, FC, and MAM: approved the final version and were accountable for all aspects of the work in ensuring that questions related to the accuracy or integrity of any part of the work are appropriately investigated and resolved.

## Conflict of Interest Statement

The authors declare that the research was conducted in the absence of any commercial or financial relationships that could be construed as a potential conflict of interest.
